# Evaluation of 3D-Printed Balls with Photopolymer Resin as Grinding Medium Used to Alternatively Reduce Warmup During Dry Milling

**DOI:** 10.3390/polym17131795

**Published:** 2025-06-27

**Authors:** Bence Borbás, Zsófia Kohod, Nikolett Kállai-Szabó, Bálint Basa, Miléna Lengyel, Romána Zelkó, István Antal

**Affiliations:** 1 Department of Pharmaceutics, Semmelweis University, Hőgyes Endre Street 7-9, 1092 Budapest, Hungary; borbas.bence@semmelweis.hu (B.B.); kohod.zsofia@semmelweis.hu (Z.K.); kallai.nikolett@semmelweis.hu (N.K.-S.); basa.balint@semmelweis.hu (B.B.); lengyel.milena@semmelweis.hu (M.L.); 2 Centre for Pharmacology and Drug Research & Development, Semmelweis University, Üllői út 26, 1085 Budapest, Hungary; zelko.romana@semmelweis.hu; 3 University Pharmacy Department of Pharmacy Administration, Semmelweis University, Hőgyes Endre Street 7-9, 1092 Budapest, Hungary

**Keywords:** particle size, dry media milling, milling ball, warmup, additive manufacturing, differential scanning calorimetry (DSC)

## Abstract

This study investigates the applicability and advantages of using additive manufacturing to moderate heat generation in dry milling. Grinding medium balls of different sizes were designed and fabricated using computer-aided design (CAD) and a stereolithographic 3D printer. Milling processes with particle size distribution and warmup measurements were employed with the printed medium balls. The results were compared with the measurements executed with conventional stainless-steel balls. Differential scanning calorimetry (DSC) was employed to evaluate the effect of the warmup of the system during the milling process. A two-variable, three-level experimental design was used for the measurements. We selected two grinding parameters considered critical: speed and time. The effect of these two independent variables on heating was examined. The results show that if printed balls are applied with the same total mass as that of metal balls, the particle size reduction is increased. The greater the number of balls used, the greater the particle size reduction. In this process, where additively manufactured milling bodies were used, the temperature of the system increased by less than when stainless-steel balls were used. The use of 3D-printed medium balls demonstrated beneficial warmup behavior.

## 1. Introduction

The ideal particle size of pharmaceutical substances including both drugs and excipients is a crucial property that determines other characteristics of the materials, their technological processibility and the performance of the pharmaceutical dosage forms [1]. To obtain the optimized particle size distribution, preprocessing with grinding or milling in dry or wet conditions is required.

The solubility of active pharmaceutical ingredient (API) powders is a key property in pharmaceutical research and development. In addition to permeability, solubility is the other attribute which is used in the Biopharmaceutical Classification System (BCS). BCS is used for assessing the pharmacokinetics of drug substances [2]. Materials in BCS class II and IV have low solubility; thus, increases in it, as seen in the case of these APIs, are a major challenge to achieving the required bioavailability and biofunctionality [3]. One of the methods that is applicable for increasing the dissolution rate is particle size reduction. As a result of grinding, the particle size distribution will become narrower than it was before the procedure; thus, the specific surface area will increase and the thickness diffusion layer around each particle will decrease, according to the Noyes–Whitney equation [4].

A conventional method with which to increase the solubility of poorly water soluble drugs is micronization, which is performed via various milling techniques such as colloid mills and jet mills [5]. Micronization is a well-established method in the formulation of dry powder inhalation systems, where the particle size of the API is decreased to below 5 μm using comminution methods [6]. Nowadays, nanonization is considered a promising tool to improve the bioavailability and solubility of drugs by formulating nanoparticles [7]. These are sub-micron-sized drug delivery systems that could be formulated with top–down (e.g., medium or bead milling; high-pressure homogenization) and bottom–up (e.g., chemical synthesis; nanoprecipitation) methods [8]. Although the particle size reduction in water-insoluble drug substances below a few microns in size demands wet milling, dry milling remains a well-established preprocessing step for larger particles of raw materials.

The particle size of materials is an important factor that determines the mixing flowing properties of powders, and it impacts the processability of solid materials.

Even the particle size of an excipient such as filler may influence homogeneity and content uniformity as well as the drug dissolution profile [9].

In addition, the flowability of particulate systems depends on the particle size [10]. Stavrou et al. investigated the influence of particle size and particle size distribution on the flowability of glass beads as a model material. The study showed that the increased particle size resulted in better flowability and a decrease in the constrain factor [11]. Particle size has an influence on bulk cohesion as well. By decreasing the particle size, bulk cohesion is increased [12]. Shah et al. studied the effect of milling time and milling temperature on bulk cohesion. The results of the study showed that by increasing the milling time, cohesion was increased, while it was found to be similar in the case of the different milling temperatures [13]. Particle size, as one of the important characteristics of a powder, has an influence on the compressibility of the powder as well. When the particle shape is constant, materials with smaller particle sizes have better compressibility properties [14].

For particle size reduction, several mechanical methods could be used, like hammer milling, jet milling and roller milling [15,16,17]. One frequently used method for reducing the particle size is milling in different ball mills. Milling balls are manufactured from different hard materials, like cast iron and steel grinding media [18], cemented tungsten carbide grinding bodies [19] such as agate, and glass- and zirconia-based media [20]. Besides these commonly used media, highly cross-linked polystyrene resin can be used as the material of the grinding bodies in the case of a medium milling process being used [21]. Guner et al. compares the breakage kinetics and temperature rise in a model material during milling with polystyrene and zirconia-grinding media during wet bed milling [22]. In a traditional ball mill, several parameters must be taken into consideration which influence the efficacy of the particle size reduction. The size and size distribution of the grinding bodies is one of the crucial parameters; by increasing the larger ball size proportion, the characteristic diameter of the grinded particles increases [23]. In the industry, using a mixture of grinding balls with different ball sizes is common to ensure an efficient grinding procedure [24]. Memarvar et al. observed that an increased number of grinding balls during milling resulted in a reduced particle size [25]. The ball-to-powder weight ratio is another important parameter that has an influence on particle size reduction. Generally, a higher ball-to-powder weight ratio results in a more effective milling process [26]. Milling time is also considered a crucial factor in particle size reduction. Canakci et al. studied the effect of milling time on particle size reduction, and the results showed that by increasing the milling time, the particle size of the powder was decreased [27]. With the increase in the rotation speed during the milling, the grinding rate increased, but the particle size limit became larger [28]. With regards to the rotation speed, two factors must be taken into consideration. The speed of the rotation has to be smaller than the critical speed because at the critical speed, the balls are positioned stationary in relation to the inner wall of the jar, preventing them from contributing to the grinding process [29].

Table 1 shows some references to previous studies in which the Retsch PM 100 planetary ball mill was used for milling.

During milling, the temperature of the system will increase since the ball collisions induce warmup during the process [38]. An elevation in the temperature can cause the degradation of heat-sensitive products [39]. In the presence of some active pharmaceutical ingredients, such as indomethacin, the rise in the temperature during the procedure causes amorphization, which can lead to a thermodynamically unstable product [40]. In the case of wet milling, a smaller final particle size can be achieved since parallel cooling is involved, making it easier to achieve; on the other hand, heat generation is a challenging risk to avoid during dry milling [39,41].

Nowadays additive manufacturing is a constantly advancing tool in the medical sciences [42]. Within additive manufacturing, numerous methods could be used to formulate drug delivery systems, like the stereolithography (SLA) method [43]. In the first step, a 3D plan of the object has to be created with a 3D design software, and prior to the printing procedure, the plan has to be sliced into several, well-defined horizontal slices in a slicing program [44]. After slicing, the g-code file, which contains the digitally prepared structure, is transported to the 3D printer, and from the pieces of information in the g-code file, the printer builds up the printlet layer-by-layer [45]. The g-code file contains the precise printing parameters as well, such as layer height, the material used for printing, the initial exposure time and the exposure time in the case of stereolithography printing. Using the SLA method, the most common source initiating photopolymerization is UV light; however, different wavelengths can be applied in the solidification of the resin to achieve a crosslinked matrix structure [46]. During the exposure of the actual layer, the liquid hardens and the platform position is optimized to provide space for the following layer [47]. The utilization of this high-precision tool enables the achievement of outstanding layer resolution in seconds [48].

In this study, the aim was to investigate the possible application of the additive manufacturing method to replace conventional milling media with more flexible resins. The 3D printing of the model dry milling balls was optional due to the sustainability cost-effectiveness of this method [49,50]. Moreover, modification of the structures manufactured by 3D printing is quick and simple to achieve, allowing for the fabrication of modified printlets on-site. A comparative evaluation was conducted, focusing on the warmup of stainless-steel balls and their supplementation with 3D-printed photopolymer resin as the model grinding medium in the conventional dry ball milling procedure for cases in which conventional materials cannot be used for grinding.

## 2. Materials and Methods

### 2.1. Materials

The commercially available 3D printing resin Prusament Resin Tough Prusa Orange (Prusa Research a.s., Prague, Czech Republic) was used to print the model grinding balls in different diameters. After the 3D printing process, isopropyl alcohol (Molar Chemicals Ltd., Halásztelek, Hungary) was used to postcure and clean the prepared objects during the postcure phase. Saccharose (d = 393 μm ± 24 μm) (Molar Chemicals Ltd., Halásztelek, Hungary), talc (Molar Chemicals Ltd., Halásztelek, Hungary) and lactose monohydrate (Molar Chemicals Ltd., Halásztelek, Hungary) were milled during the process.

### 2.2. Methods

#### 2.2.1. Design of the Grinding Balls

The 3D objects were designed with Autodesk Fusion 360 (version: Fusion 2.0.20476x86_64, Autodesk Inc., San Rafael, CA, USA), which exported the prepared structure into a stereolithography file. In the planning phase, grinding balls of different sizes (d = 25 mm; d = 20 mm; d = 10 mm) were designed. Further settings should be applied during the slicing, which is an algorithmic step in which the slicing software Prusa Slicer (version: 2.4.2, Prusa Research a.s., Prague, Czech Republic) divides the object into several, well-defined horizontal slices. The exported g-code file includes not only the coordinates the 3D printer is going to follow but all the other printing parameters which can be set, e.g., the printing mode (printing resolution), exposure times and the placements of the supports. In our experiment, the layer thickness had to be adjusted between 0.025 mm–0.1 mm and the exposure time between 1 and 60 s.

#### 2.2.2. SLS Printing

The grinding balls were printed with the Original Prusa SL1S SPEED 3D printer (Prusa Research a.s., Prague, Czech Republic). The objects were additively manufactured with a layer thickness of 0.025 mm in each layer, with support below the objects. The exposure time was 25 s in the first three layers and 6 s in the remaining layers.

The printed structures were cleaned and postcured in the Original Prusa Curing and Washing Machine (Prusa Research a.s., Prague, Czech Republic). The grinding balls were cleaned with isopropyl alcohol for 5 min, and after cleaning, 3-3 min of drying and UV postcuring were carried out.

#### 2.2.3. Densities of the Different Milling Balls

The Kern ABJ-NM/ABS-N analytical balance (Kern&Sohn GmbH, Balingen, Germany) was used to measure the mass of the stainless-steel and 3D-printed balls of different diameters to calculate the densities of the grinding bodies.

#### 2.2.4. Microscopic Images of the Milling Balls

Digital microscopic images from the grinding balls and from the saccharose before and after milling were taken with the Keyence VHX 970 Digital Light Microscope (Keyence International, Osaka, Japan).

#### 2.2.5. Weight Loss Test

A weight loss test was performed on the printed grinding balls in a Retsch 100PM (Retsch GmbH, Haan, Germany) planetary ball mill. The initial mass of the balls was 40 g in each measurement, and the mass of the printlets was measured with a Kern ABJ-NM/ABS-N analytical balance (Kern&Sohn GmbH, Balingen, Germany) before and after the process, while the mass loss was calculated from the values. The procedure time was 15 min at 300 rpm, with a 5 min break time and a 5 min interval time. The total number of revolutions was 3000.

#### 2.2.6. Grinding

##### Grinding of Saccharose and Particle Size Distribution Measurement

During the comminution of the saccharose model material, the rotation speed was 200 rpm and the milling time was 15 min, with a 5 min break time and a 5 min interval time. For comminution, the Retsch PM100 planetary ball mill (Retsch GmbH, Haan, Germany) was used. As the grinding medium used for comparison, conventional stainless-steel balls (Retsch GmbH, Haan, Germany) of the same diameter as the 3D-printed balls were used. In the case of saccharose milling, the particle size was measured via sieve analysis using a Retsch AS 200 control vibrational sieve (Retsch GmbH, Haan, Germany). The amplitude of sieving was 1.5 mm, and the sieving time was 5 min after every saccharose grinding process. Each procedure was measured under the same conditions in triplicate.

##### Structural Changes in Lactose Monohydrate During Milling Process

Lactose monohydrate was ground in a planetary ball mill. The milling time was 45 min and the milling rate was 400 rpm. During the procedure, the same amount of grinding bodies measuring the same diameter were used for the stainless-steel balls and for the printed model grinding bodies. The sizes of the used milling balls were as follows: d = 25 mm (1 piece), d = 20 mm (5 pieces), and d = 10 mm (3 pieces). After 45 min, the samples were measured with a Seiko Exstar 6000/6200 differential scanning calorimeter (DSC) (Seiko Instrument Inc., Chiba, Japan). Dispensed amounts of the 8 mg samples were placed into aluminum pans and analyzed at a 10 °C/min heating rate from a temperature of 0 °C to 200 °C. The endothermic peak related to the crystalline water loss is discussed later in the results section.

##### Warming of the Grinding Jar and Milling Balls

**Figure 1:**
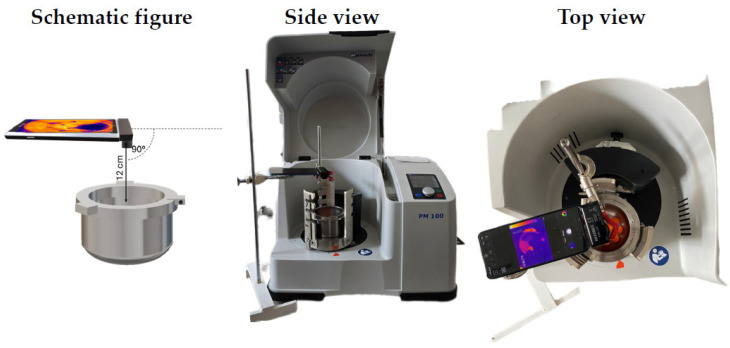
Warmup measurement setup.

The warming caused by the friction between the grinding jar and milling balls was measured with a Voltcraft WBS-220 thermal camera (Conrad Electronic international GmbH&Co.KG., Hirschau, Germany). The size of the used milling balls was d = 25 mm (1 piece), d = 20 mm (5 pieces), and d = 10 mm (3 pieces) for the original stainless-steel balls and for the 3D-printed balls. The heat camera images were taken from the same position after 15 min, 30 min and 45 min of the procedure time. The temperature of the point with the highest temperature value was measured at each sampling time and used for further analyses. The milling rates were 300, 350 and 400 rpm. Each batch was measured in triplicate. The setup of the measurement is shown in Figure 1.

##### Statistical Analysis of Warmup

TableCurve3D v4.0 (Grafiti LLC, Palo Alto, CA, USA) and software was used to perform the statistical analysis of the system’s warmup. The effect of the two independent variables on the z parameter (temperature of the system) was modelled by a polynomial function with the following formulas:

z = a + bx + cy + dx^2^ + ey^2^ + fxy (1)

where x and y are the two independent variables (x, revolutions per minute; y, process time), b and c are coefficients characterizing the main effects, d and e are the quadratic terms and parameter f describes the interaction effects. The main effects represent the average result of modifying a single factor at a time. The quadratic polynomial terms are included to examine nonlinearity. The interaction coefficients show how the response shifts when two independent factors are modified simultaneously. The two factors, as well as their levels, are shown in Table 2.

## 3. Results

### 3.1. Fabrication of the Printlets

**Figure 2:**
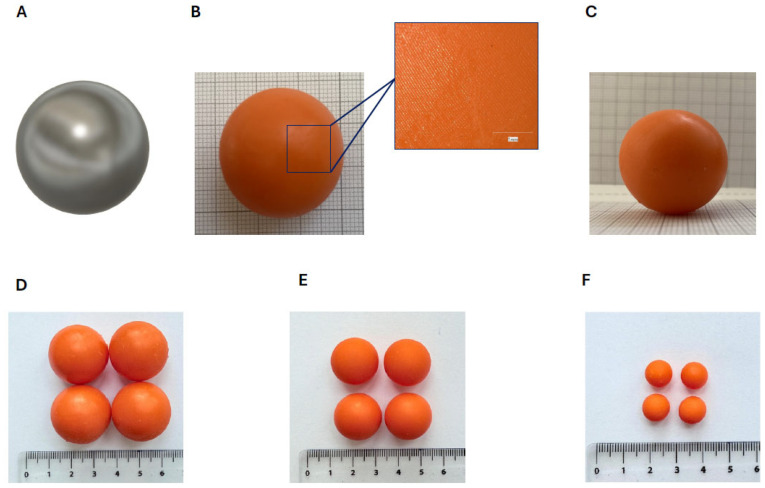
Dry milling balls fabricated via 3D printing ((**A**): CAD file of the model; (**B**): picture of the 3D-printed milling ball (top view) and digital microscopic picture of the surface; (**C**): picture of the 3D-printed milling ball (side view); (**D**): picture of the 3D-printed balls (d = 25 mm); (**E**): picture of the 3D-printed balls (d = 20 mm); (**F**): picture of the 3D-printed balls (d = 10 mm)).

Grinding balls with different diameters were printed out with an SLA printer, and after printing, UV postcuring of a defined number of the grinding balls was performed. Although several well-known printing methods could be used to print out grinding balls, the SLA method was chosen due to its high resolution rate, which also enables the additive manufacturing of smaller grinding balls. The CAD file and pictures of the fabricated balls are shown in Figure 2.

### 3.2. Densities of the Grinding Balls

The densities of the stainless-steel and 3D-fabricated grinding bodies of different sizes were calculated from the objects’ mass and volume. The results are shown in Table 3. The densities of the conventional metal balls were higher for every size than those of the additively manufactured grinding bodies.

### 3.3. Weight Loss of the Printed Milling Balls

A weight loss study of the samples with and without UV postcuring was carried out, and the weight loss, measured with an analytical balance, was negligible (0.00%).

### 3.4. Particle Size Reduction

Measurements of the postcured milling balls measuring different diameters and with different ball masses were performed in a planetary ball mill with the previously given settings, and Table 4 shows the changes in the average particle sizes for the different grinding measurements. Stainless-steel balls of the same size and aggregated ball mass were used as the grinding body for comparison.

The results show that for the balls fabricated via SLA printing, the average particle size of the milled material decreased more than it did in the stainless-steel milling bodies. Comparing the decrease in the average particle size of the saccharose, which was used during the measurements, shows that with increases in the amount of grinding balls, the average particle size decreased, as Table 4 shows. According to the particle size distribution in the different processes, the SPAN values increased when milling with the stainless-steel balls and with 3D-printed balls was carried out, which resulted in a wider particle size distribution. The 3D-printed balls (6 pcs, postcured) were the most effective at reducing particle size, but this came with a broader particle size distribution. Stainless-steel balls provided the smallest change in particle size and the narrowest distribution both before and after milling. Postcuring of 3D-printed balls appears to enhance their milling efficiency. Using more balls (6 vs. 4) led to a greater reduction in particle size.

**Figure 3:**
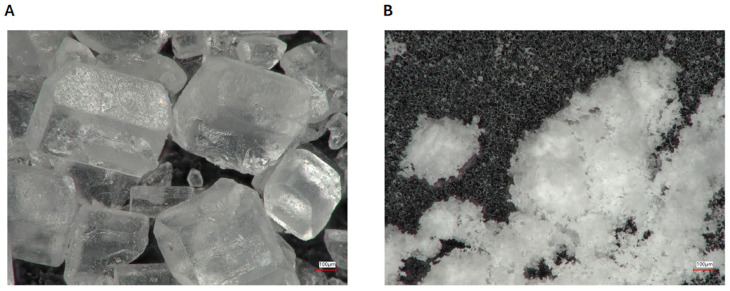
Digital microscopic images of the saccharose ((**A**) before milling and (**B**) after milling with 3D-printed balls).

Digital microscopic pictures of the saccharose particles before and after the milling performed with 3D-printed balls are shown in Figure 3.

According to the results of the previously discussed particle size distribution analyses, the grinding balls printed with the SLA technique are suitable to be used for reducing the particle size in dry milling.

When conventional stainless-steel balls are used for grinding, a non-beneficial effect must be taken into consideration. When grinding at a high rotation speed, the grinding bodies and the grinding jar heat up due to the friction between them. As a result of the heating, the ground substance may melt, which would inhibit particle size reduction. Furthermore, the heat-sensitive APIs can suffer from thermal decomposition as well. This effect, in the case of stainless-steel grinding balls, can be avoided by reducing the rotation speed, and by implementing a break time into the procedure. However, these modifications would result in the elongation of the grinding time, which is especially undesirable in the pharmaceutical industry.

### 3.5. Warmup of the System in Planetary Ball Mill

#### 3.5.1. The Effect of the Warmup on the Lactose Monohydrate Model Material

**Figure 4:**
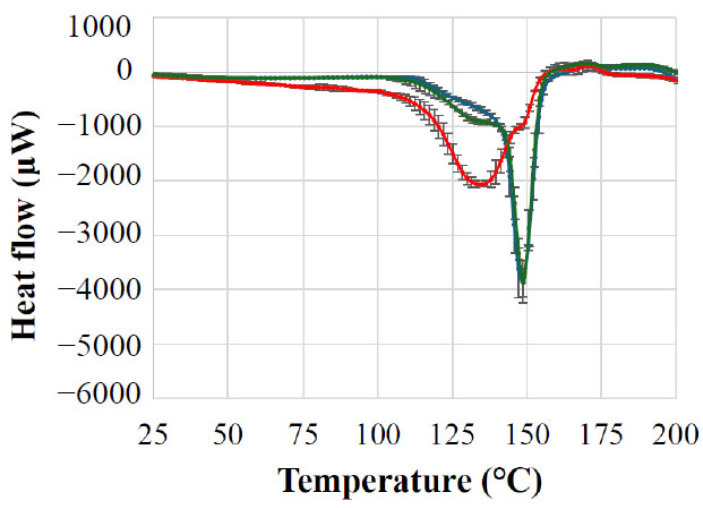
DSC analysis of the lactose monohydrate model material (blue: initial lactose monohydrate thermogram; green: thermogram after grinding with 3D-printed balls; red: thermogram after grinding with stainless-steel balls).

The effect of the warmup during the procedure was investigated with a lactose model material. After 45 min of grinding, differential scanning calorimeter measurements were made. Thermograms of raw lactose monohydrate and its milled sample after 45 min of milling with conventional stainless-steel balls and additively manufactured balls were registered. Figure 4 shows the difference in the placement and intensity of the endothermic peak in the DSC between the initial lactose monohydrate and the same material after 45 min of grinding. Original lactose monohydrate showed a sharp endothermic peak at 148.9 °C, corresponding to the loss of crystalline water [51]. The 3D-printed ball-milled lactose monohydrate retained this peak at 148.9 °C with similar sharpness, indicating no significant structural alterations from milling. The stainless-steel ball-milled lactose exhibited a broadened peak that had shifted to 134.7 °C, suggesting partial amorphization or crystal lattice destabilization due to heat-induced stress.

##### Mechanistic Implications

The temperature rise during milling with stainless-steel balls (attributed to frictional heating) likely caused the thermal degradation of the lactose’s crystalline structure. This aligns with thermocamera data showing higher temperatures (up to 30.5 °C ± 0.2 °C) for steel balls vs. 3D-printed ones. These results show that the warmup during grinding with stainless-steel balls affected the structure of the lactose model material.

#### 3.5.2. Thermocamera Measurements

**Figure 5:**
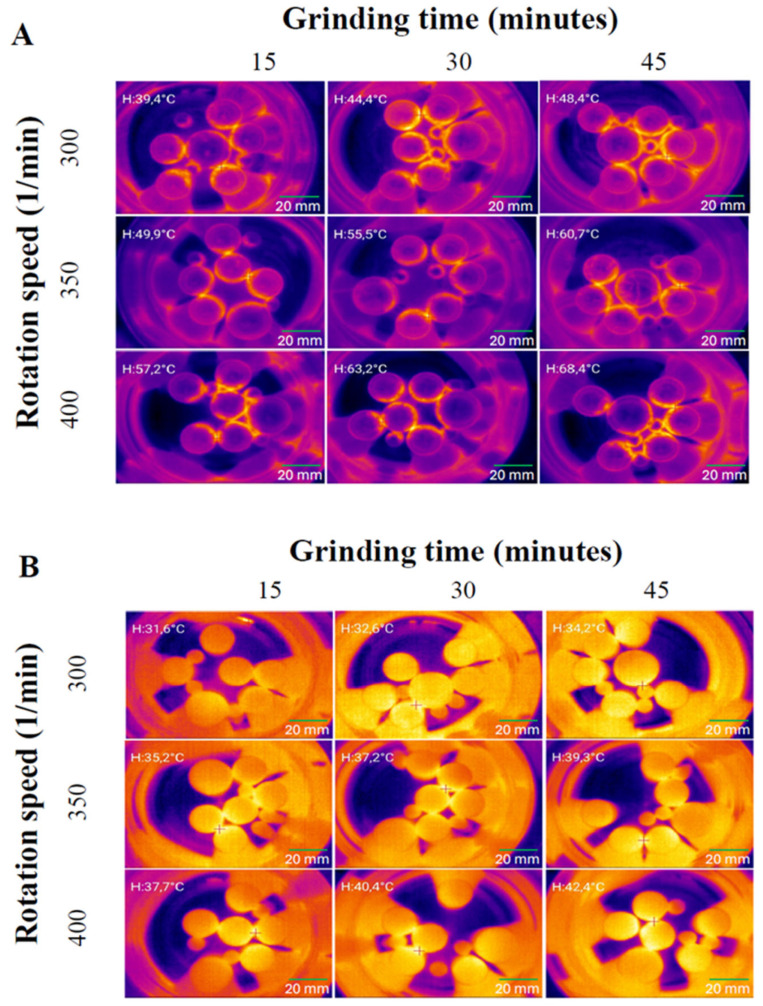
Heat camera images of the system taken in different revolutions and at different process times ((**A**) warmup of the system with traditional stainless-steel balls; (**B**) warmup of the system with 3D-printed balls).

The warmup of the balls printed with the SLA technique was measured at 300, 350 and 400 rpm speeds. The temperature changes during the procedure were visually tracked with a thermal camera within 15, 30, and 45 min of the procedure. As a comparison material, conventional stainless-steel milling bodies were used. The pictures of the heat cameras capturing images at different rotation rates and milling times are shown in Figure 5, and the temperature of the warmest point is shown in Table 5. According to the results of the measurements, the printed grinding balls heated up less than the steel balls used for comparison under the same conditions. The initial temperature of the system was 30.5 °C ± 0.2 °C. The SLA-printed balls’ temperatures were lower at every rotation speed than the values obtained for the stainless-steel balls at 15 min (shown in Figure 5 and Table 5). Generally, the temperature of the grinding bodies increased with increasing the process time and the rotation rate.

#### 3.5.3. Statistical Analysis of the Warmup

**Figure 6:**
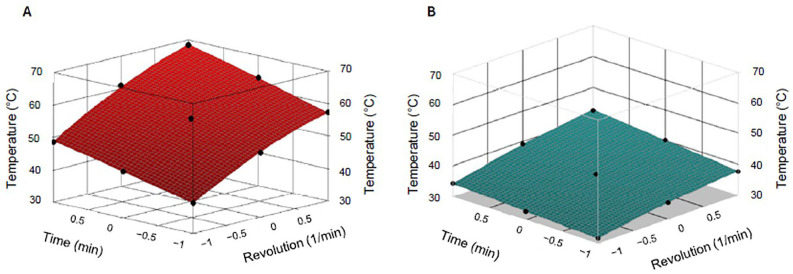
Surface plot analysis of the system’s warmup ((**A**) warmup of the stainless-steel balls; (**B**) warmup of the 3D-printed grinding media).

Surface plot analyses were performed based on the measured temperature values for both the conventional stainless-steel balls and the additively manufactured balls. Table 6 contains the calculated parameters that were used for the evaluation of the model. From the measured and calculated data, a surface plot graph was recorded (Figure 6). Parameters for which the *p* value was smaller than 0.05 were considered parameters which significantly influenced the examined parameter: the temperature of the system during the observation of the warmup. Parameter a is a constant, parameters b and c are the coefficients of the main effects (time and rate of the rotation), and parameters d, e and f are the coefficients of the side effects, which can modify the main effects.

In the case of the measurements carried out for the conventional stainless-steel balls, only parameters a, b, c and d were considered significant. Based on the evaluation of the analysis, both the time and the rate of the rotation certainly had a significant effect on the temperature of the system. The equation for the surface plot analysis of the warmup measurement carried out with metal balls is as follows:

z = 55.63 + 9.47x + 5.25y − 1.90x^2^ (2)

In the case of the measurements carried out for the 3D-printed grinding balls, parameters a, b, c, d, and f were considered significant, which means that both investigated parameters (time and rotation speed) had a significant main effect on the warmup of the grinding bodies. The equation for the surface plot analysis of the warmup measurement carried out with metal balls is as follows:

z = 37.08 + 3.65x + 1.88y − 0.62x^2^ + 0.45xy (3)

The values of the coefficients were compared between the two measurements carried out with stainless-steel and stereographically printed balls, and it was observed that the coefficients of the main effects were higher for the evaluation of the warmup of the conventional metal balls than for that of the additively manufactured ones. By comparing the main coefficients of the investigated parameters, the results showed that the increase in the time and rate of the rotation during the process elevated the temperature of the system to a greater extent in the measurement carried out with stainless-steel balls. The changes in the examined process parameters, the time and rate of the rotation, had a greater influence on the temperature of the system when the procedure was carried out with metal grinding bodies.

##### Temperature Spike Phenomenon

Mathematical Definition and Origin

The temperature spike phenomenon represents a transition from a linear to parabolic temperature increase during the ball milling process. In surface plot analysis, this manifests as significant quadratic coefficients in the polynomial regression equation: temperature = a + b (rotation speed) + c (time) + d (rotation speed^2^) + e (time^2^) + f (rotation speed x time). When the quadratic term for rotation speed (coefficient d) becomes statistically significant, it indicates that temperature no longer increases linearly but accelerates parabolically, creating the characteristic “spike” pattern.

Temperature Response Analysis: Demonstration of Temperature Spike Phenomenon in Ball Milling

The visual representation clearly demonstrates how steel balls exhibit parabolic temperature acceleration while 3D-printed balls maintain controlled, near-linear temperature increases. The comparison of the d quadratic coefficient of the rotation speed shows that the value of the coefficient is 3.06 times higher in the case of the stainless-steel balls than in the case of the 3D-printed balls.

Physical Mechanism Behind Temperature Spikes

A temperature spike occurs through a three-phase thermal process during dry ball milling [39,52]. Phase 1 involves linear heating, where the heat generation from collision friction approximately equals the heat dissipation through conduction and convection [53]. Phase 2 represents a critical transition where accumulated heat reduces the system’s heat dissipation capacity, leading to the critical point at which heat generation exceeds dissipation [39,52]. Phase 3 manifests as the temperature spike, where steel balls experience thermal runaway with a parabolic temperature rise, while 3D-printed balls maintain controlled thermal behavior due to their superior thermal properties [54,55]. The statistical analysis demonstrates that steel balls experience thermal behavior fundamentally differently to the 3D-printed alternatives. While both materials show significant linear effects due to the time and rotation speed, only steel balls exhibit the pronounced quadratic acceleration that creates the characteristic temperature spike pattern. This mathematical distinction validates the physical interpretation that steel balls undergo thermal runaway due to their higher thermal conductivity and greater frictional heating.

##### Correlation Between Statistical Analysis and Experimental Findings

Direct Evidence from DSC Analysis

The surface plot analysis predictions directly correlate with the differential scanning calorimetry results observed in the lactose monohydrate experiments. Steel balls, which exhibit significant quadratic coefficients indicating temperature spikes, caused thermal stress that shifted the endothermic peak from 148.9 °C to 134.7 °C and broadened it, indicating partial amorphization. Conversely, the 3D-printed balls, showing a controlled temperature response with interaction effects, preserved the original peak position at 148.9 °C with similar sharpness, confirming minimal thermal stress.

Thermal Camera Data Validation

The mathematical coefficients derived from surface plot analysis align precisely with thermal camera measurements throughout the milling process. Steel balls at 400 rpm reached 68.4 °C after 45 min, following a parabolic pattern predicted by the significant quadratic coefficient. In contrast, 3D-printed balls at identical conditions reached only 42.4 °C after 45 min, demonstrating the controlled temperature profile predicted based on the interaction term’s significance.

Process Performance Correlation

The surface plot findings explain the superior milling performance observed with 3D-printed balls compared to conventional steel media. The absence of dramatic temperature spikes enables continuous operation without thermal damage to materials. Lower thermal stress preserves material crystallinity while achieving better particle size reduction, as evidenced by the improved saccharose milling results.

## 4. Discussion

This article presents a concept involving an innovative and modern tool of the pharmaceutical industry, additive manufacturing, which was used in a conventional base operation, grinding. The utilization of 3D printing in this process to examine temperature, as well as dry milling, is a novel approach and represents a significant advancement in the field. Moreover, this study can form the basis for other research in that it offers an alternative method for particle size reduction. Our approach allows researchers to perform milling under wet and dry conditions without causing heating of the system; therefore, there is no need to apply break periods in this process, and the total procedure time can be decreased. Moreover, by using additively fabricated grinding balls, amorphization or degradation of the heat-sensitive active drug substances can be avoided. This could lead to a more effective, more optimized, less time-consuming and more cost-effective method that pharmaceutical industries can utilize. In the future, further developments in the use of biocompatible resin will be a huge milestone for the translation of this concept for use in the industry. The application of new materials and techniques in industrial pharmaceutical manufacturing, such as the previously mentioned polystyrene milling balls and the additively manufactured photopolymer balls, could revolutionize comminution methods.

## 5. Conclusions

In this study, we managed to fabricate grinding balls with SLA 3D printing techniques. This method was suitable for the formulation of alternative grinding media; since SLA printing has a high resolution, sphere-shaped structures of the same diameter as conventional stainless-steel grinding balls can be printed. Dry milling of saccharose with additively manufactured milling balls is proven to be an appropriate and effective method compared to milling with stainless-steel balls. It was observed that there was less warmup in the system if the grinding media were fabricated via 3D printing. The warmup of the system during the milling of APIs and excipients can cause undesirable structural changes. This phenomenon was supported by the DSC measurements of lactose monohydrate. The temperature spike identified in our surface plot analysis represents a critical physical phenomenon that determines milling success, and it is not merely a statistical artifact. The 3D-printed balls’ ability to avoid temperature spikes while maintaining effective grinding represents a significant advancement in dry milling technology. In the Supplementary Table S1 a comparison about the advantages of the 3D printed balls over the stainless-steel balls are shown. 

Future research should build upon this mathematical foundation to optimize 3D-printed grinding medium design for specific pharmaceutical applications. The correlation between statistical analysis and physical phenomena provides a framework for understanding and controlling thermal effects in mechanochemical processes. This work demonstrates how proper integration of mathematical modeling with experimental validation can advance both theoretical understanding and practical applications in pharmaceutical manufacturing.

## Figures and Tables

**Table 1:** Literature review of milling parameters in Retsch PM 100 planetary ball mill.

rpm (1/min)	Process Time (min)	Number of Balls	Diameter of Balls (mm)	Reference
300; 700	10	12	10	[30]
500	60	4	10	[31]
250	20	5	10	[32]
300	60	6; 3	10; 20	[33]
300	15	100	5	[34]
600	40	180	5	[35]
500	120	10	5	[36]
300	30	100	5	[37]

**Table 2:** Symbolized levels of the independent variables of the experimental design and kinetic parameters of the warmup.

Coded Value	Actual Values of x (1/min)		Actual Values of y (min)	
−1	300		15	
0	350		30	
+1	400		45	
	Experimental matrix		Stainless-steel balls	Printed balls
Trial No.	Coded Values of x	Coded Values of y	Temperature (°C)	Temperature (°C)
1	+1	−1	57.2 ± 0.1	37.8 ± 0.1
2	+1	0	63.3 ± 0.1	40.3 ± 0.1
3	+1	+1	68.4 ± 0.1	42.4 ± 0.1
4	0	−1	49.8 ± 0.2	35.2 ± 0.2
5	0	0	55.4 ± 0.1	37.1 ± 0.2
6	0	+1	61.0 ± 0.3	39.1 ± 0.2
7	−1	−1	39.3 ± 0.1	31.6 ± 0.1
8	−1	0	44.4 ± 0.0	32.6 ± 0.2
9	−1	+1	48.4 ± 0.1	34.4 ± 0.2

**Table 3:** The densities of the different grinding bodies (average ± relative standard deviation).

Diameter	Density of Stainless-Steel Balls (g/cm^3^)	RSD (%)	Density of 3D-Printed Balls (g/cm^3^)	RSD (%)
25 mm	0.9642	0.01	0.1491	0.75
20 mm	0.9620	0.01	0.1507	0.19
10 mm	0.9649	0.04	0.1554	0.11

**Table 4:** Average particle size and SPAN values of the saccharose ground with different milling media.

Material	Initial/Grinded	Average Particle Size (μm)	SD (μm)	SPAN	SD
Metal ball	initial	386.30	±3.42	1.01	0.03
	milled	359.89	±4.14	1.10	0.03
3D-printed balls (6 pcs with postcuring)	initial	374.02	±24.31	1.05	0.10
	milled	207.55	±12.00	1.33	0.03
3D-printed balls (4 pcs with postcuring)	initial	427.97	±37.38	0.82	0.10
	milled	307.73	±18.27	1.38	0.05
3D-printed balls (4 pcs without postcuring)	initial	382.91	±12.82	1.15	0.23
	milled	287.30	±9.79	1.26	0.14

**Table 5:** Warmup measurement of the system with stainless-steel and 3D-printed balls.

		Grinding Time (min)		
	Rotation Speed (1/min)	15	30	45
Stainless-steel balls	300	39.4 °C	44.4 °C	48.4 °C
	350	49.9 °C	55.5 °C	60.7 °C
	400	57.2 °C	63.2 °C	68.4 °C
3D-printed balls	300	31.6 °C	32.6 °C	34.2 °C
	350	35.2 °C	37.2 °C	39.3 °C
	400	37.7 °C	40.4 °C	42.4 °C

**Table 6:** Numerical results of the surface plot analysis for the warmup of the grinding balls (values in bold: significant coefficients).

		Coefficients					
Sample Name	Parameters	a	b	c	d	e	f
Stainless-steel balls	Value	**55.63**	**9.47**	**5.25**	**−1.90**	−0.35	0.53
	Std Error	0.302	0.165	0.165	0.286	0.286	0.202
	*p* > ItI	0.000	0.000	0.000	0.007	0.308	0.080
printed balls	Value	**37.08**	**3.65**	**1.88**	**−0.62**	0.08	**0.45**
	Std Error	0.158	0.086	0.086	0.150	0.150	0.106
	*p* > ItI	0.000	0.000	0.000	0.026	0.617	0.106

## Data Availability

The original contributions presented in this study are included in the article/Supplementary Material. Further inquiries can be directed to the corresponding author(s).

## Supplementary Materials

The following supporting information can be downloaded at: https://www.mdpi.com/article/10.3390/polym17131795/s1, Table S1: Enhanced Thermal Management in Pharmaceutical Applications: Comparative Advantages of 3D-Printed Prusament Tough Resin Over Stainless Steel in Mitigating Temperature Fluctuations.

## Abbreviations

The following abbreviations are used in this manuscript:

CAD	Computer-Aided Design
API	Active Pharmaceutical Ingredient
BCS	Biopharmaceutical Classification System
rpm	Revolutions Per Minute
SLA	Stereolithography
3D	Three-Dimensional
UV	Ultraviolet
DSC	Differential Scanning Calorimetry
