# Supplementary material for: Evaluation of 3D-Printed Balls with Photopolymer Resin as Grinding Medium Used to Alternatively Reduce Warmup During Dry Milling
Source: Polymers (Basel). 2025 Jun 27;17(13):1795. doi: 10.3390/polym17131795 (PMC12251631; doi:10.3390/polym17131795)
Supplement: Supplementary file 1 [file polymers-17-01795-s001.zip › polymers-3700103-supplementary.pdf]

Supplementary table S1: Enhanced Thermal Management in Pharmaceutical Applications: Comparative Advantages of 3D-Printed Prusament Tough Resin Over Stainless Steel in Mitigating Temperature Fluctuations

Advantage Category	Prusament Resin Tough Prusa Orange Benefit	Stainless Steel Ball Limitation	Pharmaceutical Impact
**Thermal Conductivity**	Low thermal conductivity acts as insulator, preventing rapid heat transfer during processing [56, 57]	High thermal conductivity (16-18 W/m^2K) causes rapid heat transfer and temperature spikes [58]	Essential for protecting heat-sensitive APIs from degradation [59]
**Temperature Spike Prevention**	Significantly reduces temperature spikes during high-energy collisions in milling processes [57]	Prone to creating sudden temperature increases due to metal-on-metal contact [60]	Critical for maintaining chemical stability and preventing polymorphic changes [61]
**Heat Generation Control**	Generates substantially less heat during mechanical operations compared to metal balls [56]	Creates significant heat generation during high-speed collision and friction [62]	Vital for processing thermolabile compounds where temperature spikes cause degradation [63]
**Thermal Stability Range**	Thermal stability up to 50°C, sufficient for most pharmaceutical processes [56, 57]	Excellent thermal stability up to 800°C but transfers heat too rapidly at lower temperatures [60]	Appropriate range for pharmaceutical processing without excessive heat transfer
**Process Temperature Control**	Provides gradual temperature changes allowing better process control and monitoring [57]	Rapid temperature fluctuations make precise control difficult in sensitive processes [62]	Important for maintaining GMP standards requiring tight temperature parameter control [61]

**Material Safety Profile**	Low odor, does not contain Bisphenol A, minimized health risks for pharmaceutical environments [56, 57]	Biocompatible but may introduce metal contamination concerns in pharmaceutical products [64]	Safer for pharmaceutical manufacturing with reduced contamination risk
**Mechanical Properties**	Good deformation resistance while maintaining thermal insulation properties [56, 65]	High hardness and wear resistance but poor thermal insulation [64, 66]	Balances mechanical performance with thermal management needs
**Energy Efficiency**	Lower material density (1.00 g/cm^3) reduces energy requirements during operation [65]	Higher density requires more energy for equivalent processing [66]	Contributes to more sustainable pharmaceutical manufacturing
**Particle Size Consistency**	More stable temperature profiles lead to consistent particle size distributions [61]	Temperature fluctuations can cause variable particle size outcomes [62]	Improves batch-to-batch consistency in pharmaceutical formulations

Reference

56. *Prusament Resin is here: Introducing our high-quality material for SLA 3D printers!* [cited 2025 06.11]; Available from: https://blog.prusa3d.com/prusament-resin-is-here-introducing-our-high-quality-material-for-sla-3d-printers_54997/.
57. *Printed Solid: Prusament Resin Tough Prusa Orange 1kg*. [cited 2025 06.11]; Available from: https://www.printedsolid.com/products/prusament-resin-tough-prusa-orange-1kg.
58. *3D printing stainless steel: hygenic and durable*. [cited 2025 06.11]; Available from: https://www.fkm.net/en/materials/metals/stainless-steel/.
59. *The Crucial Role of Temperature in Milling Efficiency & Quality*. [cited 2025 06.11]; Available from: https://z-mixer.com/the-crucial-role-of-temperature-in-milling-efficiency-quality/.
60. *Does stainless steel ball have good stability in high-temperature environment*. [cited 2025 06.11]; Available from: http://www.jinanchuanghuiguoji.com/list_38/73.html.
61. Guner, G., et al. *Predicting the Temperature Evolution during Nanomilling of Drug Suspensions via a Semi-Theoretical Lumped-Parameter Model*. Pharmaceutics, 2022. **14**, DOI: 10.3390/pharmaceutics14122840.
62. Guner, G., et al., *Analysis of heat generation during the production of drug nanosuspensions in a wet stirred media mill.* International Journal of Pharmaceutics, 2022. **624**: p. 122020 DOI: https://doi.org/10.1016/j.ijpharm.2022.122020.

63. *Pharmaceutical Milling and Sieving Technologies: Complete Guide*. [cited 2025 06.11]; Available from: https://www.s3process.co.uk/pharmaceutical-milling-and-sieving-technologies-complete-guide/.
64. *5 Materials for 3D Printed Medical Devices: Which One is Right for You?* [cited 2025 06.11]; Available from: https://www.addmangroup.com/5-materials-for-3d-printed-medical-devices-which-one-is-right-for-you/.
65. Grygier, D., et al. *Investigations into the Material Characteristics of Selected Plastics Manufactured Using SLA-Type Additive Methods*. Polymers, 2024. **16**, DOI: 10.3390/polym16111607.
66. *Guide to Stainless Steel 3D Printing*. [cited 2025 06.11]; Available from: https://www.unionfab.com/blog/2024/07/3d-printing-stainless-steel.
